# The Involvement of Bangladeshi Girls and Women in Sex Work: Sex Trafficking, Victimhood, and Agency

**DOI:** 10.3390/ijerph19127458

**Published:** 2022-06-17

**Authors:** Md. Nazmul Huda, Syeda Zakia Hossain, Tinashe Moira Dune, A. S. M. Amanullah, Andre M. N. Renzaho

**Affiliations:** 1 School of Population Health, University of New South Wales, Kensington, NSW 2052, Australia; 2 School of Health Sciences, Western Sydney University, Campbelltown, NSW 2560, Australia; t.dune@westernsydney.edu.au; 3 School of Liberal Arts and Social Sciences, Independent University, Dhaka 1229, Bangladesh; 4 ARCED Foundation, 13/1 Pallabi, Mirpur-12, Dhaka 1216, Bangladesh; 5 Translational Health Research Institute, School of Medicine, Western Sydney University, Penrith, NSW 2751, Australia; andre.renzaho@westernsydney.edu.au; 6 Faculty of Medicine and Health, University of Sydney, Camperdown, NSW 2050, Australia; zakia.hossain@sydney.edu.au; 7 Diabetes Metabolism and Obesity Translational Research Unit, Western Sydney University, Campbelltown, NSW 2560, Australia; 8 Department of Sociology, University of Dhaka, Dhaka 1000, Bangladesh; dramanullah@du.ac.bd

**Keywords:** engagement, sex work, women and girls, factors, trafficking, agency, Bangladesh

## Abstract

In Bangladesh, traffickers have trapped socially and economically marginalised girls and women and sold them into sex work. Furthermore, multiple sociocultural factors shape women’s forced and voluntary movement into sex work. However, there are limited peer-reviewed studies of how sex work operators and sociocultural and economic factors shape women’s forced and voluntary engagement in sex work in Bangladesh and worldwide. This study examines how sex work operators and various factors shape Bangladeshi women’s forced and voluntary involvement in sex work. This study used a qualitative approach by employing in-depth interviews with 10 female sex workers (FSWs) and 8 other stakeholders who work in a Bangladeshi brothel context. This study also used field notes to document how sex work operators and various factors shape women’s engagement in sex work. The interview transcripts and field notes were coded and analysed thematically. Participants’ accounts reveal two key themes about how sex work operators and sociocultural factors shape women’s engagement in sex work. Findings suggest that sex work operators (e.g., traffickers, pimps, madams, house owners) forced girls and women into sex work by putting them in situations in which they had limited power. Furthermore, various economic (poverty, limited employment opportunities) and sociocultural (rape, harassment, exploitation, divorce, limited support from family members and friends, feeling of disempowerment, desire to be autonomous) factors shaped their voluntary engagement in sex work by creating a condition of victimhood in which women felt limited agency and obligated to work for madams as bonded sex workers. However, some women supported by an FSW-led organisation had more agency than others to work and earn in the brothel area. We suggest three important strategies that are likely to benefit brothel-based women and their families, children, and the wider community.

## 1. Introduction

Sex work is not entirely legal in Bangladesh [1]. The High Court and the Ministry of Law, Justice, and Parliamentary Affairs declared sex work legal in 2000 and 2002, respectively [2]. However, while the Constitution of Bangladesh [3] has criminalised any type of sex work, the Dhaka Metropolitan Public Act [4,5] has outlawed street-based sex work in Bangladesh. This duality in the Bangladeshi legal system leads to the subsequent definition that sex work can be termed quasi-legal [6,7]. Sex work is also stigmatised and operates in adverse social circumstances in Bangladesh [6,8,9,10]. Despite this, many Bangladeshi women and girls join sex work, both voluntarily and involuntarily. According to the estimates of the United Nations Program on HIV and AIDS (UNAIDS) [11], over 140,000 Bangladeshi women and girls are engaged in commercial sex work as of February 2020; many of them entered sex work via trafficking [12].

Trafficking is commonly known as “sex trafficking” of women and girls and involves their sexual exploitation [13,14,15]. The United Nations (UN) Protocol to Prevent, Suppress, and Punish Trafficking in Persons, Especially Women and Children has defined *trafficking* for sexual exploitation (such as sex trafficking) as “any act of the recruitment, transportation, transfer, harbouring or receipt of persons, by means of the threat or use of force or other forms of coercion, of abduction, of fraud, of deception, of the abuse of power or a position of vulnerability or of the giving or receiving of payments or benefits to achieve the consent of a person or having control over another person, for the purpose of exploitation” [16]. The UN trafficking protocol involves two aspects. The first aspect is women and girls’ coerced or involuntary entry into sex work. The second aspect is girls’ underage (below 18 years of age) induction into sex work. Like the first aspect, the second aspect indicates that underage girls involved in sex work are considered “trafficked”, irrespective of whether they have consented to enter commercial sex work [13,16,17,18,19,20].

International literature has indicated trafficking as the primary reason for girls’ and women’s entry into sex work [15,21]. Studies in India [18,22,23], Iran [24], the United Kingdom [25], and the United States of America [15,26,27] identified various factors (e.g., poverty, violence, stigma associated with sex work, strong patriarchal values, discrimination in jobs, inadequate support from family members and friends, and inadequate implementation of laws) that benefitted pimps and traffickers. These factors made girls and women vulnerable to trafficking [28]. Studies also found that pimps and traffickers targeted socially and economically vulnerable girls and women who looked for unskilled and semi-skilled jobs (such as working in factories, babysitting, cooking, waitressing, and assisting sales) to support their lives and the needs of family members such as siblings and children [13,25]. Furthermore, international evidence suggests that traffickers use drugs, psychological manipulation, and violence as weapons to forcefully induct women and girls into sex work, thus limiting their agency to have control over their own body, income, and potentially safe intercourse with clients [29]. However, these studies did not focus on how sex work operators develop a strong trafficking network to manipulate girls and women into sex work and how family members act as traffickers and use girls and women as tools to earn money and engage them in sex trafficking, thus limiting their agency.

There is adequate international literature examining women’s voluntary involvement in sex work. For example, studies of foreign women in Turkey [29] and migrant women in Canada [30] reported personal (e.g., communication skills) and interpersonal (e.g., women’s connection with their fellow persons) factors shaping women’s voluntary engagement in sex work. The studies also documented some factors—poverty, high unemployment rate, colossal income inequality between men and women, limited job prospects, and poor remuneration—that influenced women’s decision to choose sex work as employment. However, there is limited peer-reviewed literature regarding girls’ and women’s deliberate induction into sex work in the context of limited-resource settings.

Specifically, there are limited peer-reviewed studies of girls’ and women’s voluntary and involuntary involvement in sex work in Bangladesh. Most studies highlighted the trafficking of girls and women based on a literature review [12,31]. Moreover, a qualitative study of migrant and trafficked women found trafficking as the main reason for women’s involvement in sex work. The study also suggested that traffickers (e.g., husbands, lovers) trapped socially and economically marginalised girls and women and sold them to the madams. Once they enter sex work, they hardly return to mainstream society [32]. A quantitative study mentioned several causes of women’s forced and voluntary movement into sex work, including poor income, limited job opportunities, and divorce [1]. In Bangladesh, girls and women find themselves in a disadvantaged position due to multiple factors, including, but not limited to, patriarchal norms, their economic dependence on males, and their underprivileged position in the gendered culture [33,34]. Such factors may contribute to their involvement in sex work [6].

However, to our knowledge, no study has discussed how various factors influence girls’ and women’s agency and entry into sex work, both voluntarily and involuntarily, from the perspectives of female sex workers (FSWs) and other stakeholders in the brothel areas in Bangladesh. In this article, we opted for the use of the terminology “female sex workers”, which, although considered pejorative by some, is consistent with the more ubiquitous and suggested terminology of the UNAIDS document [35]. This study aims to examine how sex work operators and various factors shape Bangladeshi girls’ and women’s forced and voluntary involvement in sex work. This study defines sex work operators (traffickers, men, pimps, madams, house owners) as individuals who deceive, use force, and coerce girls and women to do sexual activities, and constrain their livelihood for their benefit. The paper examines women’s and girls’ entry into sex work because it represents a point at which interventions can be potentially designed to (a) stop their forced engagement in sex work and assist them in leaving sex work (if they want to), and (b) enhance their agency, and decrease their socioeconomic vulnerabilities and contraction of emerging infectious diseases including HIV.

## 2. Materials and Methods

### 2.1. Study Design

Due to the exploratory nature of the research question and the necessity for narrative accounts, this study has a qualitative study design involving interviews and field notes. In-depth interviews were used due to their ability to provide robust insights [27,36] into contextual and sociocultural issues shaping FSWs’ forced and deliberate entry into sex work and to lead the discussion of interest on the same [37]. The primary author of this study piloted the questions with two FSWs and two other stakeholders in the Panpur brothel area and revised them in response to the local culture, nuances in language, practice, and context. Field notes were also kept to document events, contextual issues, or circumstances during ten visits for six h each to the brothels to triangulate the interview data [38]. Writing field notes was to provide rich accounts of the subjects being researched [38,39].

### 2.2. Study Sample

Between June and August 2017, we sought a purposeful sample of FSWs and other stakeholders in the brothel environment. In-depth interviews were completed with 10 FSWs and 8 other stakeholders. FSWs included women bonded and worked for madams and house owners for at least one year and later working as independent sex workers. Other interview participants included pimps (*n* = 2), madams (*n* = 2), NGO workers (*n* = 2), a police officer (*n* = 1), and a human rights advocate (*n* = 1). The study’s recruitment of participants was conducted by the first author and an NGO worker, who helped recruit FSWs and other participants with varied experiences and who were likely to provide rich data.

### 2.3. Gaining Access to the Study Site

This study was conducted among the FSW population residing and working in one of the biggest brothel areas in Bangladesh. The study site, namely the Panpur brothel area, is 150–200 km away from the capital city of Dhaka. In the brothel precinct, a local non-governmental organisation (NGO) named *Biz* has worked with FSWs and their children, madams, and pimps for more than 12 years. Before going to the Panpur brothel area, the primary author sought cooperation from *Biz* (Seed) due to their excellent reputation and rapport with FSWs, including the members of an FSW-led organisation (*Swanirvor*). *Biz* and *Swanirvor* (Self-dependent) provided the investigators with the support required for obtaining access to the study area; this ranged from renting hotel rooms to gaining access to potential participants, including FSWs, madams, pimps, and human rights advocates.

### 2.4. The Use of Pseudonyms and Rationale for Their Use

This study used pseudonyms for all study participants who participated in the in-depth qualitative interviews, including those who facilitated data collection, the FSW-led organisation, NGOs, and places (e.g., the study site) mentioned. This was because the present study contains sensitive information about local power structures, people in power, and FSWs’ human rights, which may pose threats to the organisations and participants’ lives if exposed. Accordingly, using pseudonyms may unlink their identity from a particular brothel area, sub-district, and district, thus reducing risks to their security [40].

### 2.5. Data Collection and Analysis

Interview guides were developed in collaboration with the study team based on an extensive review of existing literature [9,22,23,24,26,27,28,29,31,41,42,43]. Interviews captured information about girls and women’s voluntary and involuntary involvement in sex work in Bangladeshi brothel areas. In-depth interviews with participants took between 30 and 72 min to complete. The first author conducted all interviews in Bengali (the participants’ native language). Before collecting data, the first author received permission from the NGOs and FSW-led organisations working in the brothel area. While interviews with FSWs and madams took place in their closed private rooms in brothel houses, interviews with pimps took place in the *Swanirvor* office. Interviews with other stakeholders were held in respective offices where they felt comfortable responding. The first author wrote field notes at the end of each field activity about FSWs’ forced and voluntary involvement in sex work.

All interviews were digitally recorded using an audiotape recorder after obtaining permission from the participants. The first author and a professional transcriber transcribed all interviews in English. Draft transcripts were checked to identify any misinterpretation and inconsistencies in English transcription and made corrections where necessary. Word files of transcripts and field notes were then imported into the QSR NVivo qualitative data management software for coding. Inductive codes were developed by reading and re-reading transcripts, field notes, and repeated discussions with authors. The discrepancies in coding were subsequently reconciled through discussions with the authors. Qualitative data were analysed thematically as per the approach described by Braun and Clarke [44]. We maintained an audit trail throughout the data collection and analysis to minimise researchers’ bias.

### 2.6. Ethical Clearance

Ethical approval was obtained and granted by the Human Research and Ethics Committee (HC16544), the University of New South Wales (UNSW), Australia and the Bangladesh Medical Research Council (BMRC/NREC/2013–2016/618), the Ministry of Health and Family Welfare, Bangladesh. All participants signed consent forms and provided written consent at the beginning of each instance of data collection. Participants with no educational background provided their thumbprints on the consent forms. By signing the consent forms or providing thumbprints, participants also indicated that they were willing to have their voices audiotaped during in-depth interviews. Interview transcripts were de-identified, and pseudonyms were generated for each interview participant. Each participant received approximately BDT 250 (USD 3) for participating in this study.

## 3. Results

### 3.1. FSWs’ Background Characteristics

Table 1 summarises the sociodemographic characteristics of FSWs who participated in the in-depth interview. A total of 10 FSWs participated in in-depth interviews. FSWs were aged between 19 and 37 years, with an average age of 25. Six participants were ≤25 years old. Six FSWs had no education; one completed primary education; three participants completed secondary education. Five FSWs were currently married, three were unmarried, and two were divorced. The average number of years of FSWs’ involvement in sex work was 10 years. Their years of involvement in the sex industry ranged from 1 to 30 years. Six FSWs had regular primary partners. Seven participants had no children, while three participants had one child (Table 1). Participants’ monthly income ranged between BDT 5500 (USD 66) and BDT 50,000 (USD 602) with an average of BDT 23,650 (USD 285), which is above the average monthly income of people of Bangladesh (USD 160 approximately) [45].

### 3.2. Sociodemographic Characteristics of Other Stakeholders

In-depth interview participants also included madams, pimps, NGO workers, a police officer, and a human rights advocate. Table 2 describes the sociodemographic characteristics of other stakeholder participants in the in-depth interviews.

### 3.3. Main Findings

Qualitative findings also reveal two core themes associated with how sex work operators and various factors shaped Bangladeshi girls’ and women’s forced and voluntary engagement in sex work. These include girls’ and women’s entry into sex work by trafficking and sex work as a means of survival.

#### 3.3.1. Girls’ and Women’s Entry into Sex Work by Trafficking

Most girls and women began stories by recounting their entry into sex work by traffickers who tricked them, transported them to the brothel area, and sold them into the sex trade. Interview and field note data revealed that traffickers (e.g., pimps, house owners, landlords, and madams) developed a strong trafficking network to tempt and exploit girls and women and traffic them into sex work, sometimes by employing garment workers in the name of giving them salaried jobs in garments factories and offering a good amount of money. For example, one FSW reported that “Traffickers played tricks on girls and women in the name of giving jobs in garments factories. Traffickers kept them at their friends’ houses at first, later brought them to the brothel area clandestinely at night-time through secret routes and narrow alleys, thus selling them to madams” (Anita, FSW, age 19). Another participant reported that traffickers brought “20 girls and women on average monthly to the brothel area from various parts of Bangladesh” (Ajmol, human rights advocate). A brothel-based NGO worker supported the narratives of FSWs and a human rights advocate and described how traffickers tricked girls and women into trafficking and brought them to the brothel area.

Male and female traffickers in garments factories developed a good relationship with poor women and girls in their villages by making false promises to recruit them to garments factories in cities. Thus, traffickers trapped girls and women, sold them to madams at the cost of 30,000–50,000 Taka [361–602 USD] and finally left the brothel area with money.

(Alam, NGO worker)

Several FSW participants also indicated that their family members trapped and sold them to madams and traffickers who forced women into the sex trade, increasing their vulnerability to infectious diseases. For example, one woman commented on how her stepmother used violence as a weapon, forcefully inducted her into sex work at home, increased her vulnerability to HIV/STI transmission, and eventually she became a commercial FSW in the Panpur brothel area:

After my biological mother’s death, my stepmother sometimes allowed strangers [clients] into our house and sent them to my room for entertainment. My stepmother used to torture me a lot if I declined a client. Such coerced sex work increased my risk of contracting various diseases and infections... She [stepmother] had a good connection with a madam. Once she sold me to her [madam] for a price of 20,000 Taka [241 USD], who brought me to this *para* [brothel area].

(Sabu, FSW, age 22)

Another FSW explained that her helpless and poor economic situation created a condition of victimhood in which her biological aunt sold her to a trafficker to earn money:

I was parentless; so, I was staying with my biological aunty. As she [my aunty] thought her household expenses increased due to my stay at her house, one day aunty forcefully sold me to a trafficker. Then, my madam bought me from the trafficker, and kept me at a house to work and earn for her [madam].

(Anita, FSW, age 19)

Several participants reported that husbands also sold their wives to traffickers and put them in a situation in which wives had no choice but to sell sex to earn money for their husbands. For example, Hani recounted how husbands negotiated with madams and sold their wives, and completed necessary formalities to live on sex work money earned by their wives, leaving no earnings for their wives:

Some people [husbands] sold their wives to madams and negotiated with them to earn money through their wives’ sex work. After bringing their wives, husbands ordered them [their wives] to enrol in sex work and collect licenses needed to engage in sex work. While leaving the brothel area, they [husbands] encouraged them, saying, ‘I am always with you’. Thus, the women were inducted into sex work, and their husbands earned money. What was left for the women at last?

(Hani, madam and house owner, age 40)

These narratives were substantiated by a human rights advocate who has promoted FSWs’ rights for 30 years and an FSW who highlighted a situation in which women had the limited agency to avert trafficking:

Sometimes husbands sold their wives to madam and left the place [brothel area] with money. They [wives] could not do anything.

(Ajmol, human rights activist, age 50)

Once, I witnessed a husband selling his wife to a madam. The woman had limited scope [capacity] to do anything then because she was not aware of it [trafficking]; because she believed in him [her husband].

(Hasina, FSW, age 21)

Hasina’s narratives also indicated that women became the victims due to their limited awareness about trafficking and trust in partners. Similarly, patterns emerging from the data indicated that boyfriends also acted as traffickers to trade their girlfriends into sex work, and girlfriends trafficked by their boyfriends exercised limited agency due to their trust in partners. Piklu, a pimp who brought women and girls and sold them to madams, acknowledged: “Many boyfriends tricked girls and women into love, made them feel overwhelmed and trusted, intending to traffic them. Then, they [men] detained and raped them [girls and women] and eventually sold them to the madams. Girlfriends had nothing to do since they did not realise it [trafficking]” (Piklu, Pimp, age 40). Another pimp, Rana, supported Piklu’s explanations and narrated how trafficked girls and women were forced into sex work, making them vulnerable to work and earn for madams: “After being sold into sex work, if girls were not agreed to work and earn for madams, they were beaten up by their madams” (Rana, Pimp, age 52). An FSW confirmed Rana’s narratives: “No one can do anything if a woman is trafficked and sold to a madam” (Odhora, FSW, age 25).

Interview and field note data also indicated that girls’ and women’s induction into sex work was pushed by various factors such as poverty, oppression, and rape. For instance, Hasina explained how her experience of harassment by village men and trust in a friend created a sense of victimhood and led her to be tricked and trafficked into sex work:

Some men in the village were not good people; they used to pass bad comments about me. That is why, I left my parents and village out of anger. I stayed at an aunt’s house for 10–15 days when I came to Dhaka. Once, I went out with my friend to see her friend’s newborn baby. Suddenly, the girl [my friend’s friend] took my phone number and asked me to contact and meet her for a job. I trusted her like a sister and met her. However, that girl [trafficker] sold me to a madam here [brothel area] for 50,000 Taka [602 USD].

(Hasina, FSW, 21)

Hasina’s descriptions also indicated that her poor socioeconomic conditions prompted her to look for a job, thus being tricked and trapped into trafficking. Another FSW illustrated how her neighbour deceived and sexually exploited her by promising her a job in Dhaka city and eventually sold her to madam:

I was brought here [brothel area] in the name of giving me a job in Dhaka city. Before bringing here, I was raped by my neighbour on the roadside. Then, he [neighbour] brought me here [the brothel area] and sold to a madam. I was devastated and found no way to go.

(Israt, FSW, age 30)

Furthermore, some women were introduced into sex work primarily due to their economic dependence on husbands and vulnerable socioeconomic conditions. For instance, Lisa reported how her experience of violence and poor economic situations put her in a condition of being trafficked into sex work by her friend, who took advantage of her economic vulnerabilities:

I never intended to come here [brother area]. No one jumps in fire by themselves. In my married life, my husband tortured me. He would not let me eat or dress. Then, I tried to get a job and visited my friend who said that she would give me a job in a garments factory. However, instead of giving me a job, she (my friend) sold me to a madam. Thus, I was bound to write my name as a sex worker and stay here [brother area] with her [madam].

(Lisa, FSW, age 20)

Patterns emerging from the data suggested that many poor girls and women were unaware of their trafficking. Most traffickers told women that they would be transported to Dhaka for jobs. Traffickers had taken advantage of women’s vulnerable economic situation, limited employment opportunities, inadequate awareness, and no or limited education. For example:

A girl promised me that she would employ me as a housemaid. Instead of giving me the job, she sold me to a madam for 60,000 Taka [723 USD]. Initially, I was not aware that I was trafficked into sex work. As time passed, I realised that I was sold to a madam.

(Odhora, FSW, age 25)

Likewise, another FSW reported being sold into sex work through a false promise of giving her a job. For instance, Hritu described how she was deceived and forced into sex work.

A pimp brought me to this *para* [brothel precinct] through false and exaggerated promises of a prosperous life in a city. He sold me to a madam for a price of 3000 Taka [36 USD] and ran away. I did not come here to do sex work at my own will. (Hritu, FSW, age 21)

From these findings, it appeared that trafficked girls and women loved and trusted their family members, friends, and partners in the pursuit of a job or a better life. However, traffickers created a situation in which trafficked girls and women were not aware of trafficking and being tricked into sex work. Finding limited or no support from families and other sources, they worked and earned for madams in the brothel area.

#### 3.3.2. Sex Work as a Means of Survival

In contrast to trafficking, several participants, including FSWs, framed sex work as a means of survival in the event of adverse social situations. Women’s experiences of poverty, rape, forced prostitution, divorce, and false marriage prompted them to come to the brothel area and engage in sex work. For example, one woman reported being enticed into marriage by her boyfriend but was instead raped by him and his accomplices. This incident led her to visit the brothel area and engage in sex work voluntarily to earn her livelihood. Another FSW reported that she felt bound to join sex work after failing to find alternative work outside the brothel area. The following statement clearly articulates these sociocultural aspects of FSWs’ choice of sex work as a means of survival. It comes from a participant that was working as a police officer in the brothel area:

Women such as those who are raped, divorced, separated, and widowed and those in a vulnerable economic position and cannot lead their normal life in the society take shelter in the brothel area and chose sex work.

(Bablu, police officer)

Interview findings also revealed that women arriving in the brothel precinct found very few options to work and earn independently. This is primarily because madams and/or house owners rarely allow these women to rent rooms or houses to engage in sex work and thus earn money for survival. The limited options, or complete lack of options, for renting a room or a house places newly arrived women in the context of working for madams and/or house owners. Consequently, some women were not trafficked into sex work but became bound to working and earning for madams and/or house owners. This was the case for Camelia, who felt obligated to choose to join sex work for survival after her divorce. However, she became bound to work and earn for her madam for a year as no madam and/or house owner would allow her to rent a room or house to perform sex work and thus earn money independently:

No one brought me here [to the brothel area]; I came here of my own will. After failing to rent a room, I started working for my *sardarni* [madam]. I worked for her [madam] as I needed to feed myself; I did not have any other way or anyone to take care of me. What else could I have done? So, I had to choose this means [sex work] for my own livelihood. I also needed to feed my family.

(Camelia, FSW, age 30)

Camelia’s narrative also suggests that some women moved into sex work as a means of choosing employment. Once entering sex work, women felt obligated to choose sex work as their sole form of employment and work under madams as bonded sex workers for a specific period to survive and meet their family members’ subsistence and educational needs, including parents, siblings, or children. The quote also indicates that FSWs’ obligations to work under madams and earn for them for a certain time limit their agency to work independently. This was substantiated by a woman who worked as a sex worker for thirty years and madam and house owner for eight years and bonded four girls during the interviews.

Until they [girls] become independent sex workers, they work for madams for about 1–5 years. Madams manage houses for a living and bear all expenses like feeding, house rent, paying for their cosmetic luxuries and giving them some money. Thus, girls get used to living and working in the brothel area, considering their survival and family needs. Once they become independent sex workers, they earn and live on their own.

(Shuha, madam and house owner, age 45)

Shuha’s explanations indicate that independent sex workers had more agency over their living and earnings than bonded sex workers. Field note data suggested that girls and women who voluntarily joined sex work as a means of survival could work independently to survive and meet their needs in the past. Interview data suggested that an FSW-led organisation, “*Amrao Pari*” (We can do), played a crucial role in renting houses for girls who chose sex work as a form of employment. This was supported by a quote of an FSW who described that girls and women supported by the organisation could exercise more agency in terms of renting houses and doing sex work:

Once, an organisation called ‘*Amrao Pari*’ managed newly arrived girls who entered sex work. If they agreed to work here [brothel area], she could stay, and the organisation managed rooms/houses for them. If they [girls] wanted to leave sex work, they were sent to their home. Women’s joining and leaving sex work depended on their [women] choice because they felt supported by the organisation.

(Reshma, FSW, age 27)

Overall, trafficking and broader sociocultural and other contextual issues contributed to FSWs’ moving into sex work in the brothel area and choosing sex work as a means of survival.

## 4. Discussion

Globally, a growing body of literature has examined women’s entry into sex work due to trafficking [26,31,46] and their experience of sociocultural factors that shaped their entry into sex work [24,28,30,41,47]. However, these studies do not explain how madams and house owners developed a strong trafficking network to trick girls and women into sex work and how family members forcefully induct women into sex work for economic gains. Furthermore, these studies do not adequately explain how various sociocultural factors influence girls’ and women’s agency and entry into sex work, both voluntarily and involuntarily, from the perspectives of FSWs and other stakeholders in the context of resource-limited settings. Our study extends previous research through a qualitative analysis of how sex work operators (e.g., traffickers, pimps, madams, house owners) and various factors (e.g., poverty, limited employment opportunities, rape, harassment, exploitation, divorce, limited support from family members and friends, feeling of disempowerment, desire to be autonomous, etc.) shaped Bangladeshi girls’ and women’s involuntary and voluntary involvement in sex work from the perspectives of FSWs and other stakeholders in the brothel areas in Bangladesh. Our study underscores the importance of the necessity for implementing existing anti-trafficking laws, introducing safety net programs, and re-establishing an FSW-led organisation for optimising FSWs’ livelihood and creating an enabling environment in which women feel supported in the brothel area and beyond.

Consistent with previous studies [20,26,31,42], our study demonstrated that although multiple sociocultural factors shaped FSWs’ pathways into sex work, trafficking remained the primary mode of girls’ and women’s entry into sex work in Bangladesh. Traffickers approached women, touted the promises of good jobs in the city, transported them to the brothel area, and sold them into sex work for economic gains. All of these practices associated with the trafficking of women are a gross violation of all articles (see trafficking protocol) of the UN’s trafficking protocol [16]. The way women were deceived, forced, and exploited undoubtedly characterises the trafficking of women, and this intentional exploitation is indicated in the UN protocol’s definition of trafficking. Bangladesh is not yet a signatory of the trafficking protocol to prevent, suppress, and punish the trafficking of women and children [48]. Although Bangladesh is trying to prevent the trafficking of women through the “Human Traffic Deterrence and Suppression Act 2012”, it has failed to adequately protect trafficked women from exploitation [48]. As such, trafficked women in Bangladesh are more likely to experience exploitation in brothel settings than non-trafficked women [32,42]. As the exploitation of women tends to limit their agency [13,14], it is also likely that this limited agency decreases condom use and increases vulnerability to transmission of various contagious diseases, including HIV and sexually transmitted infections (STIs) [22,28,29]. Therefore, Bangladesh must emphasise the proper implementation of existing trafficking laws to disrupt trafficking networks, reduce the exploitation of women, enhance their agency, and thus minimise their socioeconomic vulnerabilities and health risks such as HIV and STIs.

The current study also found that girls and women were often tricked and trafficked into sex work by trusting traffickers (e.g., family members, relatives, neighbors, and friends) or not understanding traffickers’ tricks and deceitful actions. This was potentially due to their inadequate awareness of trafficking and limited/no education [6]. Furthermore, in a patriarchal society such as Bangladesh, girls and women are generally considered passive, submissive, and unassertive [34]. Culturally, women are attached to raising children and economically dependent on males. Such a status of women in the Bangladeshi society makes them vulnerable to economic and sociocultural obstacles such as exploitation, poverty, divorce, and abuse, making them subject to be tricked and trafficked by trusting others [6]. Therefore, the government and other policymakers should increase awareness about women’s trafficking in sex work. The findings of our study are expected to provide critical insights from gender perspectives as to girls’ and women’s standing in gender expectations in the Bangladeshi society.

Our study also indicated that some women were not trafficked into sex work. Instead, they chose to engage in sex work of their own volition, albeit because their choices were constrained. Sometimes, women’s experiences of poverty, rape, divorce, and false marriage outside the brothel area shaped their decisions and made them feel obligated to engage in sex work. Gradually, women chose sex work as a viable form of employment, both for empowering themselves and their survival and meeting the subsistence needs of their families. We did not find any study in Bangladesh to compare our findings with. However, our findings are similar to existing studies beyond Bangladesh [24,28,30,41]. These studies suggest that although various factors, including poverty/economic needs, job loss, higher income in sex work, limited support from family members, attraction to a luxurious life, exploitation, abuse, and rape, motivated women joining sex work, they chose sex work as employment. Women who voluntarily choose sex work as a form of employment may have more agency than those coerced into sex work [49]. This signals that women have some power to develop a sense of autonomy over their sexuality [7] and make favourable decisions about their choice of occupation [24]. Our findings suggest introducing safety net programs for these women and girls who felt obligated to join sex work due to their experiences of sociocultural factors outside the brothel area. At the same time, we suggest that involvement in sex work can be considered a viable strategy for girls and women who choose sex work as a practical response to meet their financial and familial needs and empower themselves.

Our study’s findings also indicated that an FSW-led organisation could play an important role in creating agency for the girls and women trapped without choice and enabling those girls and women who decided to choose sex work as a form of employment in renting houses, earning, and leaving on their own. These findings are novel in Bangladesh, and similar contexts, suggesting no peer-reviewed study has highlighted the role of an FSW-led organisation in enhancing trafficked and non-trafficked women’s agency to make their own decisions regarding their lives and sexualities, including joining sex work, working, and earning for their survival. Such agency may facilitate girls and women to establish control of their lives and sexuality, enhance their ability to lead their lives, directly negotiate with clients regarding safe sex behaviours, and decrease their vulnerability to HIV [22]. It is also important to regulate the whole sex work industry to promote sex work and women’s human rights.

Our findings also emphasise the re-establishment of an FSW-led organisation that can enhance trafficked and non-trafficked FSWs’ survival strategy and create an enabling brothel environment in which FSWs feel supported. Our pro-sex work perspective points to the fact that girls and women have a right to life and livelihood and choose sex work as a form of employment [50]. Such a pro-sex work approach may also indicate that women can make their own decisions regarding choosing commercial and intimate partners and using condoms, thus potentially contributing to reducing HIV and STIs and violence against them [6]. This pro-sex work view also brings us to the quasi-legal status of sex work in Bangladesh [6], which may increase women’s vulnerability to abuse and exploitation [50]. Our pro-sex work positioning may enhance women’s rights to life and livelihoods and give them a voice against abuse and exploitation.

### Strengths and Limitations

A major strength of this study is that its participants (e.g., FSWs) represent one of the most at-risk populations in Bangladesh. Information related to women’s involvement in sex work that this study has generated may be useful for further research into similar settings and may have policy implications. Second, the current study provides an understanding of key research areas that have received little or no attention in previous research exploring the sociocultural factors which shape women’s engagement in sex work. Third, this study employed a range of stakeholders, including FSWs, madams, pimps, police, NGO workers, and human rights advocates, which provides diverse perspectives regarding FSWs’ forced and voluntary involvement in sex work.

However, there are several limitations of this study. This study potentially does not ensure the credibility of the findings using a technique such as member checking [51,52,53]. Second, our study was restricted to two pathways of women’s entry into sex work only: trafficking (coerced) and non-trafficking (economic) pathways. It did not include other pathways to sex work, including the drug and pleasure pathways. Third, this study had limited data on condom use and associated infectious diseases. Fourth, the study’s sample size is another limitation, and findings are based on 10 FSWs and eight other stakeholders. Specifically, only one participant each from two occupations (e.g., police administration and human rights advocate) and one of the brothel areas in Bangladesh were chosen for in-depth interviews. This was due to limited availability of other stakeholder participants and time and financial constraints. These limitations indicate that further studies need to be conducted involving more brothel areas in Bangladesh, with a larger sample of FSWs and adequate participants from occupations such as police and human rights advocates. Another area for further research would be how women’s forced engagement in sex work increased their vulnerability to infectious diseases, including HIV and STIs. This will provide a better understanding of the nexus between women’s involvement in sex work in the brothel environment and their vulnerability to infectious diseases in Bangladesh.

## 5. Conclusions and Policy Implications

This study examined how sex work operators and various factors shaped Bangladeshi girls’ and women’s coerced and voluntary involvement in sex work from the perspectives of FSWs and other stakeholders in the brothel areas in Bangladesh. Our study indicated that sex work operators (e.g., traffickers, men, madams, house owners) forced girls and women into sex work by putting them in a situation where they felt limited power. We also found that various economic and sociocultural factors—poverty, limited employment opportunities, rape, harassment, exploitation, divorce, limited support from family members and friends, feeling of disempowerment, desire to be autonomous, etc.—shaped their voluntary engagement in sex work by creating a condition of victimhood in which women felt limited agency and obligated to work for madams as bonded sex workers. However, some women supported by an FSW-led organisation had more agency than others to work and earn in the brothel area. Our study’s findings have three critical implications. First, our findings underscore the necessity for implementing existing anti-trafficking laws to stop the trafficking of girls and women into sex work. The proper implementation of existing laws has the potential to destroy trafficking networks, safeguard women from trafficking and exploitation, enhance agency, and reduce their vulnerability to HIV. Additionally, the government should increase awareness about women’s trafficking to ensure that no girls or women are trafficked for sex work without their consent. Second, we suggest safety net programs for women and girls who felt obligated to join sex work due to their experiences of adverse social circumstances. Sex work can also be recognised as a viable form of employment for women and girls intending to choose sex work and engage in this business of their volition to satisfy their and their families’ needs, thus empowering themselves. Finally, our findings emphasise regulating sex work and the re-establishment of an FSW-led organisation that can enhance trafficked and non-trafficked FSWs’ survival strategy and create an enabling brothel environment in which brothel-based girls and women make their own decisions regarding sexuality, living, and earning. These strategies will likely benefit the vulnerable and poor women and girls and their families, children, and the wider community.

## Figures and Tables

**Table 1:** Background characteristics of FSWs at the time of interview (*n* = 10).

Background Characteristics	*n* (%)
Age	
19–25 years	6 (60.0)
>25 years	4 (40.0)
Level of formal education	
No formal education	6 (60.0)
1–5 years of education	1 (10.0)
6 and above years of education	3 (30.0)
Marital status	
Never married	3 (30.0)
Currently married	5 (50.0)
Divorced/separated/widowed/previously married	2 (20.0)
Number of children	
No children	7 (70.0)
1 child	3 (30.0)
Length of involvement in sex work in exchange for money/services/goods	
1–5 years	2 (20.0)
6–10 years	3 (30.0)
>10 years	5 (50.0)
Monthly total income (USD)	
USD 24–180	3 (30.0)
>USD 180	7 (70.0)

**Table 2:** Sociodemographic characteristics of stakeholder participants in the in-depth interviews.

Stakeholder Participants	Pseudonyms	Sociodemographic Characteristics
Madams	Shuha	Female, 45 years, no formal education, unmarried, Muslim, sometimes offered prayer, worked as a madam for 8 years, number of children 4, monthly income USD 145, only brothel-based source of income.
	Hani	Female, 40 years, no formal education, unmarried, Muslim, never offered prayer, worked as a madam for 12 years, number of children 2, monthly income USD 361, only brothel-based source of income.
Pimps	Rana	Male, 52 years, no formal education, married, Muslim, sometimes offered prayer, no girlfriend, monthly income USD 120, brought 4 commercial clients each day, sometimes tricked girls and women into trafficking, also worked as a boarding house caretaker, stayed in own his house at night.
	Piklu	Male, 40 years, no formal education, married, Muslim, sometimes offered prayer, no girlfriend, monthly income USD 108, brought 5 commercial clients each day, sometimes tricked girls and women into trafficking, also worked as a rickshaw puller, stayed in his own house at night.
NGO workers	Sahajada	Male, 49 years, completed higher secondary education, married, Muslim, sometimes offered prayer, 17 years as an NGO worker.
	Alam	Male, 65 years, completed Master’s degree, married, Muslim, sometimes offered prayer, 37 years as an NGO worker.
Human rights advocate	Ajmol	Male, 50 years, completed Master’s degree, married, no religion/Humanism, sometimes offered prayer, 30 years as a human rights advocate.
Police	Bablu	Male, 37 years, completed Master’s degree, married, Muslim, sometimes offered prayer, worked as a police officer in the Panpur brothel area for 1 year, monthly income USD 506, entered brothel area 20 times each week for leisure and doing duty.

## Data Availability

Data can be obtained from the corresponding author upon a reasonable request.

## References

[1] Hossain Q., Zaman A., Roy A. (2015). Lives of Brothel Based Sex Workers in Khulna. Bangladesh. Int. J. Soc. Work. Hum. Serv. Pract..

[2] Ministry of Law Justice and Parliamentary Affairs (2002). Mapping Exercise on HIV/AIDS-Law, Ethics, and Human Rights.

[3] (2010). Government of Bangladesh: Constitution of the Peoples Republic of Bangladesh. http://bdlaws.minlaw.gov.bd/act-367.html.

[4] Huq S., Cornwall A., Correa S., Jolly S. (2013). Confronting our prejudices: Women’s movement experiences in Bangladesh. Development with a Body: Sexuality, Human Rights and Development.

[5] Sex Workers Network Bangladesh The Status of Sex Workers in Bangladesh 2016. https://www.google.com/url?sa=t&rct=j&q=&esrc=s&source=web&cd=&cad=rja&uact=8&ved=2ahUKEwiYw8fq_7H4AhXnZWwGHZYCDC4QFnoECAUQAQ&url=https%3A%2F%2Ftbinternet.ohchr.org%2FTreaties%2FCEDAW%2FShared%2520Documents%2FBGD%2FINT_CEDAW_NGO_BGD_25667_E.pdf&usg=AOvVaw2wBmoQ8Riiimsj_catkQf1.

[6] Huda M.N. (2019). A mixed-Methods Study of Sex Work and the HIV Prevention Environment for Female Sex Workers in Bangladesh. Ph.D. Thesis.

[7] Huda M.N., Rawstorne P., Dune T.M., Stephenson N. (2022). How sex work operators constrain living and working environments of female sex workers in Bangladesh: A qualitative study. The 24th International AIDS Conference.

[8] Shewly H.J., Nencel L., Bal E., Sinha-Kerkhoff K. (2020). Invisible mobilities: Stigma, immobilities, and female sex workers’ mundane socio-legal negotiations of Dhaka’s urban space. Mobilities.

[9] Amanullah A.S.M., Huda M.N., Sabet D.M., Rahman T., Dhaka S.A. (2012). Commercial sex and vulnerability to HIV infection: A study among the children of sex workers (SWs) in Tangail brothel. Sex Workers and Their Children in Bangladesh: Addressing Risks and Vulnerabilities.

[10] Amanullah A.S.M., Huda M.N. (2012). Study on the Situation of Children of Sex Workers in and around Daulatdia Brothel.

[11] Joint United Nations Programme on HIV/AIDS (UNAIDS) (2020). AIDSinfo: Country factsheets Bangladesh 2018. http://aidsinfo.unaids.org/.

[12] (2015). Biswas: Human Trafficking Scenario in Bangladesh: Some Concerns. Int. J. Humanit. Soc. Sci. Stud..

[13] Cimino A.N. (2017). Sex work and adult prostitution: From entry to exit. Handbook of Behavioral Criminology.

[14] Doezema J. (2002). Who gets to choose? Coercion, consent, and the UN Trafficking Protocol. J. Gender Dev..

[15] Gerassi L. (2015). From exploitation to industry: Definitions, risks, and consequences of domestic sexual exploitation and sex work among women and girls. J. Hum. Behav. Soc. Environ..

[16] United Nations Office on Drugs Crime (2006). Travaux Préparatoires of the Negotiations for the Elaboration of the United Nations Convention Against Transnational Organized Crime and the Protocols Thereto.

[17] United Nations (2000). Protocol to Prevent, Suppress and Punish Trafficking in Persons, especially Women and Children, Supplementing the United Nations Convention against Transnational Organized Crime. https://www.ohchr.org/en/instruments-mechanisms/instruments/protocol-prevent-suppress-and-punish-trafficking-persons.

[18] George A., Sabarwal S. (2013). Sex trafficking, physical and sexual violence, and HIV risk among young female sex workers in Andhra Pradesh, India. Int. J. Gynecol. Obstet..

[19] Sandy L. (2009). ‘Behind Closed Doors’: Debt-Bonded Sex Workers in Sihanoukville, Cambodia. Asia Pac. J. Anthropol..

[20] Servin A.E., Brouwer K.C., Gordon L., Rocha-Jimenez T., Staines H., Vera-Monroy R.B., Strathdee S.A., Silverman J.G. (2015). Vulnerability factors and pathways leading to underage entry into sex work in two Mexican-US border cities. J. Appl. Res. Child. Inf. Policy Child. Risk.

[21] Dahal P., Joshi S.K., Swahnberg K. (2015). ‘We are looked down upon and rejected socially’: A qualitative study on the experiences of trafficking survivors in Nepal. Glob. Health Action.

[22] Devine A., Bowen K., Dzuvichu B., Rungsung R., Kermode M. (2010). Pathways to sex-work in Nagaland, India: Implications for HIV prevention and community mobilisation. AIDS Care.

[23] Swendeman D., Fehrenbacher A.E., Ali S., George S., Mindry D., Collins M., Ghose T., Dey B. (2015). “Whatever I have, I have made by coming into this profession”: The intersection of resources, agency, and achievements in pathways to sex work in Kolkata, India. Arch. Sex. Behav..

[24] Karamouzian M., Foroozanfar Z., Ahmadi A., Haghdoost A.A., Vogel J., Zolala F. (2016). How sex work becomes an option: Experiences of female sex workers in Kerman, Iran. Cult. Health Sex..

[25] Dodsworth J. (2015). Pathways into Sexual Exploitation and Sex Work: The Experience of Victimhood and Agency.

[26] Deshpande N.A., Nour N.M. (2013). Sex trafficking of women and girls. Rev. Obstet. Gynecol..

[27] Cobbina J.E., Oselin S.S. (2011). It’s not only for the money: An analysis of adolescent versus adult entry into street prostitution. Sociol. Inq..

[28] Goldenberg S., Silverman J.G., Engstrom D., Bojorquez-Chapela I., Strathdee S.A. (2014). “Right Here is the Gateway”: Mobility, sex work entry and HIV risk along the Mexico–US Border. Int. Migr..

[29] Kaya O., Erez E. (2018). Migration, agency, and the sex industry: Practitioners’ perspectives on foreign sex workers in Turkey. Int. J. Offender Ther. Comp. Criminol..

[30] Goldenberg S.M., Krüsi A., Zhang E., Chettiar J., Shannon K. (2017). Structural determinants of health among im/migrants in the indoor sex industry: Experiences of workers and managers/owners in Metropolitan Vancouver. PLoS ONE.

[31] Amin M.R., Sheikh M.R.I. (2011). Trafficking women and children in Bangladesh: A silent Tsunami of Bangladesh. J. Econ. Sustain. Dev..

[32] Siddiqua Y., Matin W.M., Sabet D., Ahmad S. (2012). Risks and vulnerabilities of migrant and trafficked women living in Jessore: A qualitative study. Sex Workers and Their Children in Bangladesh: Addressing Risks and Vulnerabilities.

[33] Sultana H. (2020). Migration, Trafficking, Sex Work and Constrained Choices: Gender and Sustainable Development in Bangladesh. Urban Spaces and Gender in Asia.

[34] Huda M.N., Ahmed M.U., Uddin M., Hasan M.K., Uddin J., Dune T.M. (2022). Prevalence and Demographic, Socioeconomic, and Behavioral Risk Factors of Self-Reported Symptoms of Sexually Transmitted Infections (STIs) among Ever-Married Women: Evidence from Nationally Representative Surveys in Bangladesh. Int. J. Environ. Res. Public Health.

[35] Joint United Nations Programme on HIV/AIDS (UNAIDS) (2015). UNAIDS Terminology Guidelines 2015.

[36] Dumay J.S. (2011). The qualitative research interview. Qual. Res. Account. Manag..

[37] Berry R.S., Guest G., Namey E.E., Mitchell M.L. (2013). Collecting data by in-depth interviewing. Collecting Qualitative Data: A Field Manual for Applied Research.

[38] Phillippi J., Lauderdale J. (2018). A Guide to Field Notes for Qualitative Research: Context and Conversation. Qual. Health Res..

[39] Hellesø R., Melby L., Hauge S. (2015). Implications of observing and writing field notes through different lenses. J. Multidiscip. Healthc..

[40] Scarth B.J. (2016). Bereaved participants’ reasons for wanting their real names used in thanatology research. Res. Ethics.

[41] Ingabire M.C., Mitchell K., Veldhuijzen N., Umulisa M.M., Nyinawabega J., Kestelyn E., Van Steijn M., Van De Wijgert J., Pool R. (2012). Joining and leaving sex work: Experiences of women in Kigali, Rwanda. Cult. Health Sex..

[42] Rosy S.Y. (2013). Trafficking in Women in Bangladesh: Experiences of Survivors and Challenges to Their Reintegration.

[43] Zhang X., Mao L., Aggleton P., Zhang J., Jing J., Cui J., Zhao R., Ren J., de Wit J. (2015). Factors associated with women’s entry into the sex industry: Findings from interviews conducted with female sex workers in Chinese detention centres. Sex. Health.

[44] Braun V., Clarke V. (2006). Using thematic analysis in psychology. Qual. Res. Psychol..

[45] World Bank (2020). GDP per Capita (Current US$)—Bangladesh. https://data.worldbank.org/indicator/NY.GDP.PCAP.CD?locations=BD.

[46] Andrade Rubio K.L. (2021). La demanda de migrantes indocumentadas en la industria del sexo de Nevada. Ciencia Técnica y Mainstreaming Social.

[47] McClarty L.M., Bhattacharjee P., Blanchard J.F., Lorway R.R., Ramanaik S., Mishra S., Isac S., Ramesh B., Washington R., Moses S. (2014). Circumstances, experiences and processes surrounding women’s entry into sex work in India. Cult. Health Sex..

[48] US Department of State (2017). Trafficking in Persons Report.

[49] Dodsworth J. (2014). Sexual exploitation, selling and swapping sex: Victimhood and agency. Child Abus. Rev..

[50] Ahmed S., Sabet D., Ahmad S. (2012). Legitimacy, Legality and Consent: Sex Workers and Their Children in Bangladesh. Sex Workers and Their Children in Bangladesh: Addressing Risks and Vulnerabilities.

[51] Miller C. (2000). Determining validity in qualitative inquiry. Theory Pract..

[52] Mays N., Pope C. (2000). Qualitative research in health care: Assessing quality in qualitative research. BMJ Br. Med. J..

[53] Murphy F., Yielder J. (2010). Establishing rigour in qualitative radiography research. Radiography.

